# Pharmacodynamic Modeling of Anti-Cancer Activity of Tetraiodothyroacetic Acid in a Perfused Cell Culture System

**DOI:** 10.1371/journal.pcbi.1001073

**Published:** 2011-02-03

**Authors:** Hung-Yun Lin, Cornelia B. Landersdorfer, David London, Ran Meng, Chang-Uk Lim, Cassie Lin, Sharon Lin, Heng-Yuan Tang, David Brown, Brian Van Scoy, Robert Kulawy, Lurdes Queimado, George L. Drusano, Arnold Louie, Faith B. Davis, Shaker A. Mousa, Paul J. Davis

**Affiliations:** 1 Signal Transduction Laboratory, Ordway Research Institute, Albany, New York, United States of America; 2 Flow Cytometry Core Facility, Ordway Research Institute, Albany, New York, United States of America; 3 Emerging Infections and Pharmacodynamics Laboratory, Ordway Research Institute, Albany, New York, United States of America; 4 Department of Otorhinolaryngology, University of Oklahoma Health Sciences Center, Oklahoma City, Oklahoma, United States of America; 5 Pharmaceutical Research Institute, Albany College of Pharmacy and Health Sciences, Rensselaer, New York, United States of America; 6 Albany Medical College, Albany, New York, United States of America; The University of Tennessee Health Science Center, United States of America

## Abstract

Unmodified or as a poly[lactide-co-glycolide] nanoparticle, tetraiodothyroacetic acid (tetrac) acts at the integrin αvβ3 receptor on human cancer cells to inhibit tumor cell proliferation and xenograft growth. To study *in vitro* the pharmacodynamics of tetrac formulations in the absence of and in conjunction with other chemotherapeutic agents, we developed a perfusion bellows cell culture system. Cells were grown on polymer flakes and exposed to various concentrations of tetrac, nano-tetrac, resveratrol, cetuximab, or a combination for up to 18 days. Cells were harvested and counted every one or two days. Both NONMEM VI and the exact Monte Carlo parametric expectation maximization algorithm in S-ADAPT were utilized for mathematical modeling. Unmodified tetrac inhibited the proliferation of cancer cells and did so with differing potency in different cell lines. The developed mechanism-based model included two effects of tetrac on different parts of the cell cycle which could be distinguished. For human breast cancer cells, modeling suggested a higher sensitivity (lower IC50) to the effect on success rate of replication than the effect on rate of growth, whereas the capacity (Imax) was larger for the effect on growth rate. Nanoparticulate tetrac (nano-tetrac), which does not enter into cells, had a higher potency and a larger anti-proliferative effect than unmodified tetrac. Fluorescence-activated cell sorting analysis of harvested cells revealed tetrac and nano-tetrac induced concentration-dependent apoptosis that was correlated with expression of pro-apoptotic proteins, such as *p53*, *p21*, *PIG3* and *BAD* for nano-tetrac, while unmodified tetrac showed a different profile. Approximately additive anti-proliferative effects were found for the combinations of tetrac and resveratrol, tetrac and cetuximab (Erbitux), and nano-tetrac and cetuximab. Our *in vitro* perfusion cancer cell system together with mathematical modeling successfully described the anti-proliferative effects over time of tetrac and nano-tetrac and may be useful for dose-finding and studying the pharmacodynamics of other chemotherapeutic agents or their combinations.

## Author Summary

Clinical treatment protocols for specific solid cancers have favorable response rates of 20%–25%. Cancer cells frequently become resistant to treatment. Therefore, novel anti-cancer drugs and combination regimens need to be developed. Conducting enough clinical trials to evaluate combinations of anti-cancer agents in several regimens to optimize treatment is not feasible. We showed that tetrac inhibits the growth of various cancer cell lines. Our newly developed *in vitro* system allowed studying the effects of tetrac over time in various human cancer cell lines. Our mathematical model could distinguish two effects of tetrac and may be used to predict effects of other than the studied dosage regimens. Human breast cancer cells were more sensitive to the effect on success of replication than the effect on growth rate, whereas the maximum possible effect was larger for the latter effect. Nanoparticulate tetrac, which does not enter into cells, had a larger effect than unmodified tetrac. The combinations of tetrac and resveratrol, tetrac and cetuximab (Erbitux), and nano-tetrac and cetuximab showed approximately additive effects. Our *in vitro* perfusion system together with mathematical modeling may be useful for dose-finding, translation from *in vitro* to animal and human studies, and studying effects of other chemotherapeutic agents or their combinations.

## Introduction

Tetraiodothyroacetic acid (tetrac) is a deaminated thyroid hormone analogue that binds to the integrin αvβ3 receptor for thyroid hormone [1], [2]. Tetrac inhibits binding of agonist L-thyroxine, T_4_, and 3,5,3′-triiodo-L-thyronine, T_3_, to the integrin on cultured cells [1], blocking nongenomically-initiated effects of T_4_ and T_3_ on signal transduction pathways [2]–[4]. Tetrac also has actions at the receptor independent of T_4_ and T_3_, including inhibition of cancer cell proliferation [2]–[4] and angiogenesis [5], [6]. The integrin is largely expressed on tumor cells and dividing blood vessel cells [7]. Acting at the surface of cancer cells, tetrac alters expression of differentially-regulated cancer cell survival pathway-relevant genes. These include upregulation of expression of pro-apoptotic BcL-x short form [3] and other pro-apoptotic genes [8], upregulation of anti-angiogenic *thrombospondin 1* and downregulation of several families of anti-apoptotic genes [8], [9]. Covalently bound to the exterior of a nanoparticle, tetrac does not gain access to the cell interior—where it may have thyromimetic activity [10]—and has biological activity at the integrin receptor similar to that of unmodified tetrac, but with desirable effects on cell survival pathway genes that differ from the parent thyroid hormone analogue [8], [9].

To further characterize *in vitro* the anti-proliferative pharmacodynamics (PD) of tetrac and nanoparticulate tetrac (nano-tetrac), with and without other chemotherapeutic agents, we developed a perfusion bellows cell culture system based on a perfusion (‘hollow fiber’) model. The hollow fiber model was modified by two co-authors (AL, GLD) from a previous system that explored antibiotic pharmacodynamics [11]. The hollow fiber model and perfusion bellows cell culture system allow simulation of concentration-time profiles (pharmacokinetics) expected in humans in an *in vitro* system and study of the effects over time (PD) of anti-infective and anti-cancer agents *in vitro* [12], [13]. Such *in vitro* systems in combination with mathematical modeling can support translation from *in vitro* to animal models and human clinical trials. The developed pharmacodynamic model describes the full time course of drug effects at various concentrations simultaneously and may be used to predict the effects of other than the studied dosage regimens.

We report here that tetrac and nano-tetrac inhibit cancer cell proliferation on a concentration-dependent basis that can be cell line-specific. Harvesting cancer cells from the perfusion bellows cell culture system permits fluorescence-activated cell sorting (FACS) analysis of cell cycle, and for apoptosis, quantitation of specific pro-apoptotic and anti-apoptotic gene expression by RT-PCR or microarray. Unmodified tetrac and nano-tetrac were tested in this model system for anti-proliferative efficacy alone or in combination with two other anticancer agents, the stilbene resveratrol [14], and commercially-available anti-epidermal growth factor receptor (EGFR) monoclonal antibody (cetuximab, Erbitux). Additive effects were obtained with combinations of tetrac or nano-tetrac and those other chemotherapeutic agents. We report studies in several human cancer cell lines to infer the applicability of the model and to confirm, not surprisingly, that there are dose-dependent differences in responses of specific cell lines to the chemotherapeutic agents tested.

Overall our aim to develop a mechanism-based pharmacodynamic model that characterizes the action of tetrac on human cancer cells in a newly developed perfusion bellows cell culture system was well achieved as described in the present report.

## Results

### Action of tetraiodothyroacetic acid (tetrac) on cancer cell proliferation

**Figure 1:**
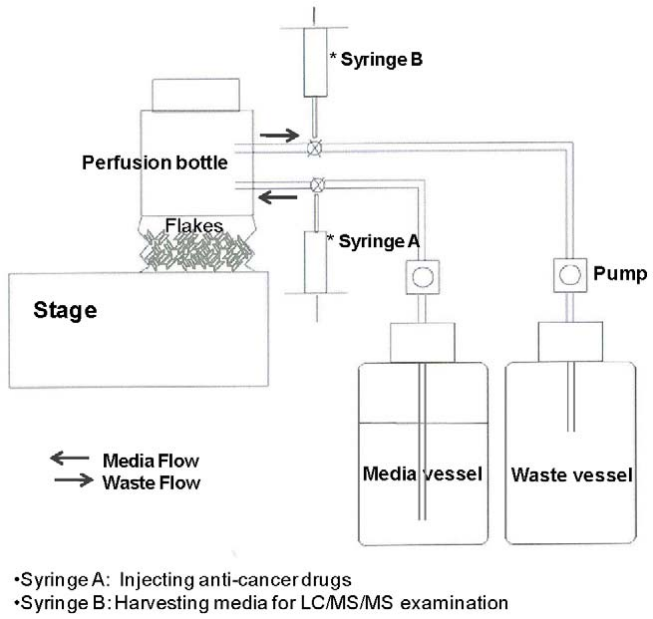
The perfusion bellows cell culture system. Cells of cancer cell lines of interest are grown on plastic flakes suspended in a flow-through, bellows-agitated system that allows for homogeneous exposure of cells to drug/drug metabolite buffer solutions and air. The system permits frequent sampling of cells for viability. The direction of each arrow indicates the direction of influx and efflux of the culture medium. Components of system are not drawn to scale.

The pharmacodynamics of tetrac as an anti-proliferative agent against human cancer cell lines were examined in the perfusion bellows cell culture system depicted in Fig. 1. Stability of tetrac in the culture system was determined by LC/MS/MS. Without cells, 75% of the original tetrac concentration was detected after 24 h incubation in medium with 10% FBS at both room temperature and 37°C. Tetrac decayed by 12% when incubated with cells at 37°C, indicating that tetrac is relatively stable in the perfusion bellows cell culture system.

**Figure 2:**
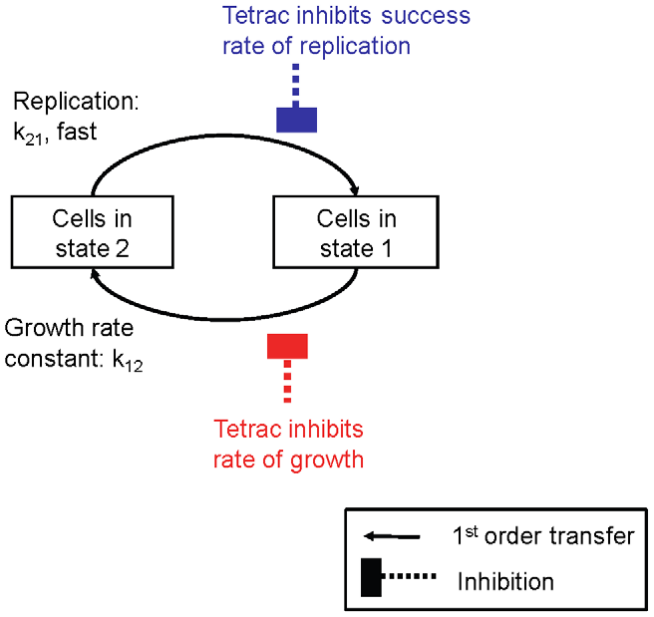
Diagram of the mathematical model. This model assumes two populations of cells in different states of the cell cycle: cells that are preparing for replication (State 1) and cells that are immediately ‘pre-replication’ (State 2). Cells transition from State 1 to State 2 by a first-order growth rate constant, while replication from State 2 to State 1 is assumed to be rapid.

**Figure 3:**
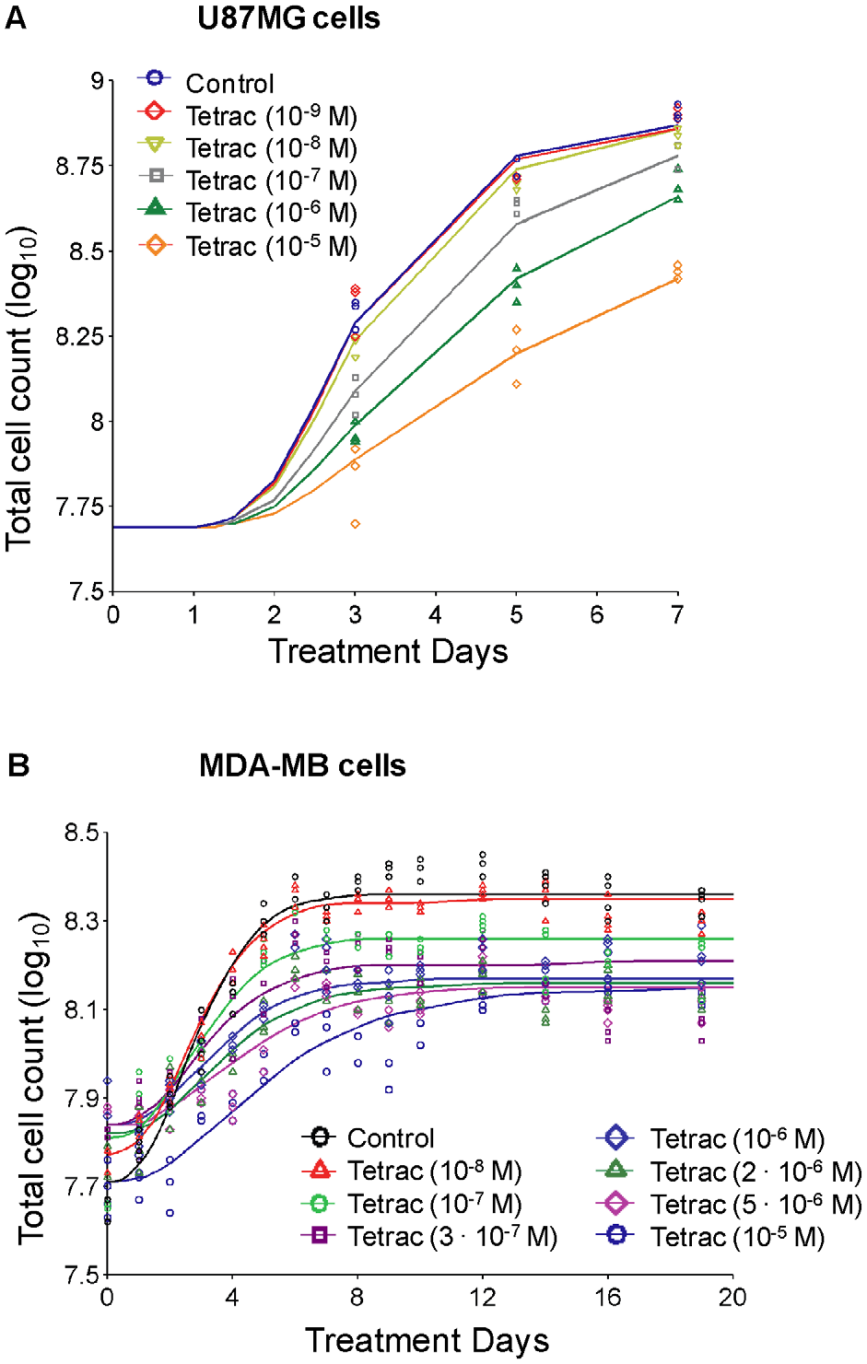
Tetrac suppresses proliferation of glioma and breast cancer cells. U87MG cells (**A**) and MDA-MB-231 cells (**B**) were treated with different constant concentrations of tetrac (10^−9^ to 10^−5^ M). Cells were harvested and counted at intervals as indicated. Total cell numbers from each treatment were used as indicators of tetrac-induced anti-proliferation. Multiple observations at each time point are multiple cell counts from one experiment.

At first tetrac induced anti-proliferation of cancer cells was studied in the non-perfusion system. Human glioblastoma U87MG cells were treated with different tetrac concentrations (10^−9^–10^−5^ M) for 7 d, with daily replenishment of tetrac. A model incorporating effects of tetrac on both growth rate and success of replication (Fig. 2) adequately described the time course of cell counts as shown by comparison of the model fitted lines to the observed data (Fig. 3A). Tetrac caused a concentration-dependent reduction in U87MG cell proliferation (Fig. 3A), where 10^−9^ M was least effective, and 10^−8^ and 10^−7^ M caused 15% and 28% decreases in cell counts compared to untreated cells after treatment for 7 d (Fig. 3A). Both effects on growth rate and probability of successful replication were required to describe inhibition of cell proliferation of U87MG cells, as shown by a statistically significant (p<0.01) difference in NONMEM's objective function.

Parameter estimates suggested U87MG cells being more sensitive to tetrac's effect on growth rate than to the effect on success of replication (IC50k<IC50R, Table 1). However, the capacity (i.e. the largest possible effect at very high concentrations of tetrac) was higher for the effect on success of replication than the effect on rate of growth (ImaxR>Imaxk). For this model the cell count on day 0 was fixed based on the number of seeded cells. From simulation-estimation experiments (50 replicates, very rich sampling, additive error on log_10_-scale = 0.1) the median bias was −4% for Imaxk, +25% for IC50k, +0.4% for ImaxR, and −2% for IC50R, using the MC-PEM algorithm in S-ADAPT. When the same bootstrap datasets plus 50 additional ones were run in NONMEM, the median bias was −2% for Imaxk, +16% for IC50k, −2% for ImaxR and −7% for IC50R (nominal results from NONMEM shown in Table 1). Bootstrap results for the actual sampling times in the experiments were similar to those from the rich design (Table 1). The two effects were therefore estimable and distinguishable, both under ideal conditions and in the actual sampling schedule which was employed in our experiment. For additional model evaluation, S-ADAPT with the MC-PEM algorithm was also used to estimate the parameters from the observed data. The S-ADAPT results for ImaxR and IC50R were within 15% of the results from NONMEM, while Imaxk was 22% lower and IC50k was 70% higher than the results from NONMEM. All other parameters were within 40% of their NONMEM estimates. The satisfactory agreement of parameter estimates from two completely different algorithms suggests adequate estimability of the model parameters.

**Table 1:** Parameter estimates and their uncertainty for effects of tetrac formulations on proliferation of cancer cells (all results from NONMEM).

			Effect on rate of growth		Effect on success of replication		
Cell line	Formulation		Imaxk	IC50k (µM)	ImaxR	kiR (day^−1^)	IC50R (µM)
U87MG	Tetrac	Estimate	0.57	0.047	0.92	-	47.4
		P50, P10–P90	0.55	0.054	0.91	-	44.2
		(rich sampling)	0.52–0.59	0.036–0.072	0.78–0.97	-	37.3–53.2
		P50, P10–P90	0.58	0.047	0.91	-	42.4
		(actual times)	0.54–0.62	0.027–0.077	0.79–0.98	-	35.7–55.2
U87MG	Nano-tetrac	Estimate	0.33	0.0001^a^	1.0^b^	0.92	0.074
		P50, P10–P90	0.33	0.0001^a^	1.0^b^^,^ c	0.91	0.075
		(rich sampling)	0.31–0.35	-	-	0.84–0.98	0.057–0.097
		P50, P10–P90	0.39	0.0001^a^	1.0^b^^,^ c	0.88	0.055
		(actual times)	0.32–0.46	-	-	0.76–1.1	0.026–0.126
MDA-MB	Tetrac	Estimate	0.85	5.1	0.20	-	0.087
		P50, P10–P90	0.85	5.0	0.20	-	0.091
		(rich sampling)	0.84–0.86	4.6–5.4	0.19–0.20	-	0.083–0.099
		P50, P10–P90	0.85	4.8	0.19	-	0.075
		(actual times)	0.81–0.91	3.7–6.4	0.18–0.20	-	0.059–0.098
MDA-MB	Nano-tetrac	Estimate	1.0	6.3	1.0^b^	1.2	0.0086
		P50, P10–P90	1.0^b^^,^ c	6.5	1.0^b^^,^ c	1.2	0.0085
		(rich sampling)	-	5.6–7.5	-	1.1–1.3	0.0075–0.010
		P50, P10–P90	1.0^b^^,^ c	10.3	1.0^b^^,^ c	1.1	0.0069
		(actual times)	-	5.0–131	-	0.93–1.2	0.0039–0.013

MDAMB: MDA-MB-231; P50, P10, P90: median, 10% and 90% percentile from bootstrap runs.

The IC50 estimates for nano-tetrac are hypothetical concentrations that assume all of the tetrac bound to the nanoparticle is available for binding to the integrin receptor.

The bootstraps were run both with a rich sampling design (n = 50 datasets) and the sampling times used in the actual experiments (n = 100 datasets).

a Fixed to 0.0001 µM as it was estimated very low and the lowest studied concentration was 0.001 µM.

b ImaxR at time = 0 d; ImaxR decreases with time (*ImaxR* = *ImaxR_0_* · *e^−kiR · time^*), possibly due to functional adaptation of cells or the presence of two or more subpopulations with different sensitivities to tetrac.

c Fixed for the bootstrap.

-not applicable.

In addition, estrogen receptor-α (ERα)-negative human breast cancer MDA-MB-231 cells (MDA-MB) were treated with 7 different concentrations of tetrac (10^−8^ to 10^−5^ M) for 19 d and total cell counts determined every 1–2 d (Fig. 3B). A model with effects on both rate of growth and success of replication (Fig. 2) adequately described the data (Fig. 3B). Parameter estimates from NONMEM are shown in Table 1. The parameter estimates suggest a higher sensitivity for the effect on probability of successful replication (IC50R<IC50k, Table 1) and a larger capacity of the effect on growth rate (Imaxk>ImaxR). Simulation-estimation experiments (50 replicates, additive error on log_10_-scale = 0.1) showed a median bias of +3% for Imaxk, +9% for IC50k, −2% for ImaxR, and +6% for IC50R, using the MC-PEM algorithm in S-Adapt. For 100 datasets in NONMEM the median bias was +0.5% for Imaxk, −0.4% for IC50k, +0.5% for ImaxR and +4% for IC50R. The bootstrap results based on the actual sampling design which was also rich were similar (Table 1). As for the action of tetrac on U87MG cells, both effects were therefore estimable and distinguishable. In S-ADAPT (MC-PEM), the parameter estimates based on the observed data were within 20% of those from NONMEM for 5 parameters and were within 50% for the other 3 parameters.

Although tetrac had a growth-suppressive effect late in the treatment period, it may also have a proliferative effect on cancer cells (results not shown here). This is thought to reflect access of the agent to the cell interior where it is a modest thyroid hormone agonist (thyromimetic) [9], [10], [15] rather than an inhibitor, as it is exclusively at the cell surface receptor.

### Anti-proliferative effects of nano-tetrac in cancer cells

**Figure 4:**
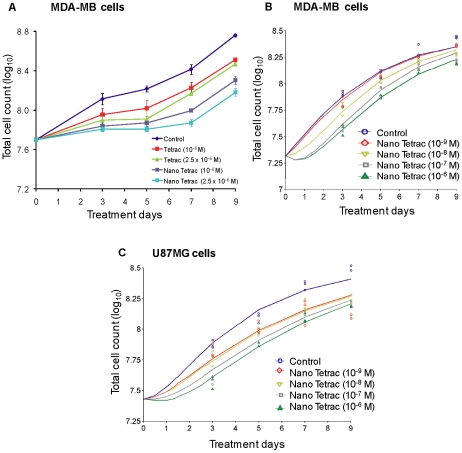
Tetrac and nano-tetrac suppress cell proliferation. (**A**) MDA-MB-231 cells were treated with two constant concentrations of either tetrac or nano-tetrac (10^−6^ and 2.5×10^−6^ M) and cells harvested at the time points indicated. Total cell numbers from each treatment were used as indicators of tetrac- or nano-tetrac-induced anti-proliferation. Nano-tetrac appeared more effective. (**B**) MDA-MB-231 cells were treated with constant concentrations of 10^−9^ to 10^−5^ M nano-tetrac and cells harvested at the time points indicated. Total cell counts from each treatment were used as indicators of anti-proliferative effect. Model-fitted lines are shown. (**C**) The effect of nano-tetrac (10^−9^–10^−6^ M) on proliferation of U87MG glioma cells is shown. As with MDA-MB-231 cells in (**B**), a concentration-dependent effect was obtained. Multiple observations at each time point represent results from 3 repeat experiments. Error bars in Fig. 4A are standard deviations from 3 experiments.

To prevent uptake of tetrac by cancer cells, it was reformulated as poly[lactide-co-glycolide] nanoparticle [8], [9], [16]. MDA-MB cells were treated with constant concentrations of 10^−6^ and 2.5×10^−6^ M tetrac or nano-tetrac for 9 d. Results indicate that the anti-proliferative effect of nano-tetrac in MDA-MB cells is greater than that of unmodified tetrac (Fig. 4A). MDA-MB cells were also treated with 4 different concentrations of nano-tetrac (10^−9^ to 10^−6^ M) for 9 d (Fig. 4B). Based on mathematical modeling, the sensitivity of the MDA-MB cells for the nano-tetrac effect on probability of successful replication was considerably higher than for the effect on growth rate (IC50R = 0.0086 µM, IC50k = 6.3 µM, Table 1), while the capacity was similar for both effects (Imaxk = 1.0, ImaxR = 1.0 at time = 0). Simulation-estimation experiments (50 replicates, additive error on log_10_-scale = 0.1) showed a median bias of +12% for IC50k, −0.8% for kiR, and +2.5% for IC50R, using the MC-PEM algorithm in S-ADAPT. For 100 datasets in NONMEM the median bias was +4.0% for IC50k, −2.5% for kiR, and −1.3% for IC50R. The bootstrap results based on the actual sampling design are shown in Table 1.

The anti-proliferative effect of nano-tetrac was also concentration-dependent in human glioblastoma U87MG cells. At a nano-tetrac concentration of 10^−9^ M, cell number was reduced by 36% (control vs. 10^−9^ M nano-tetrac = 1.45×10^8^±3.3×10^7^ vs. 2.28×10^8^±1.9×10^7^, average±S.D.) after 7 treatment days (Fig. 4C). Modeling suggested a higher sensitivity for the effect on rate of growth (IC50k<IC50R, Table 1) and a higher capacity for the effect on replication (Imaxk < ImaxR). Both IC50k and IC50R were lower for nano-tetrac than unmodified tetrac in U87MG cells indicating a higher sensitivity to nano-tetrac. For both MDA-MB and U87MG cells, the model includes a decrease in ImaxR of nano-tetrac over time in order to adequately describe the observed cell counts. Such a decrease in ImaxR might potentially be due to functional adaptation or presence of subpopulations with different sensitivities to tetrac. Simulation-estimation experiments (50 replicates, additive error on log_10_-scale = 0.1) showed a median bias of +2.1% for Imaxk, −2.8% for kiR, and +5.7% for IC50R, using the MC-PEM algorithm in S-Adapt. For 100 datasets in NONMEM the median bias was +1.5% for Imaxk, −1.5% for kiR, and +1.3% for IC50R. The bootstrap results based on the actual sampling design are shown in Table 1. The individual measurements presented as symbols in Fig. 4B and 4C are the results from 3 repeat experiments, i.e. one data point represents one experiment at each time point. The error bars in Fig. 4A are standard deviations from 3 experiments.

**Figure 5:**
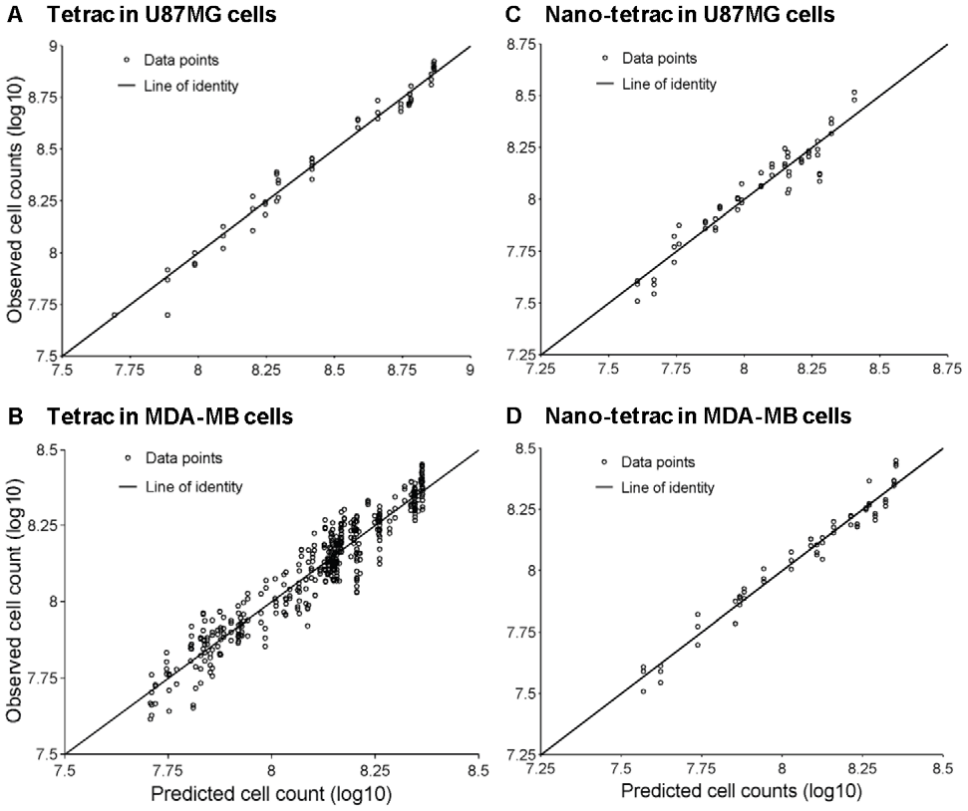
Goodness of fit plots. Observed versus predicted cell counts from NONMEM for the effect of unmodified tetrac on (**A**) U87MG and (**B**) MDA-MB-231 cells and the effect of tetrac nanoparticles on (**C**) U87MG and (**D**) MDA-MB-231 cells.

The plots of observed versus predicted cell counts are presented in Fig. 5 for unmodified and nano-tetrac in U87MG and MDA-MB cells and show that the time course of cell counts was adequately described.

### Apoptosis in tetrac-treated MDA-MB cells

**Figure 6:**
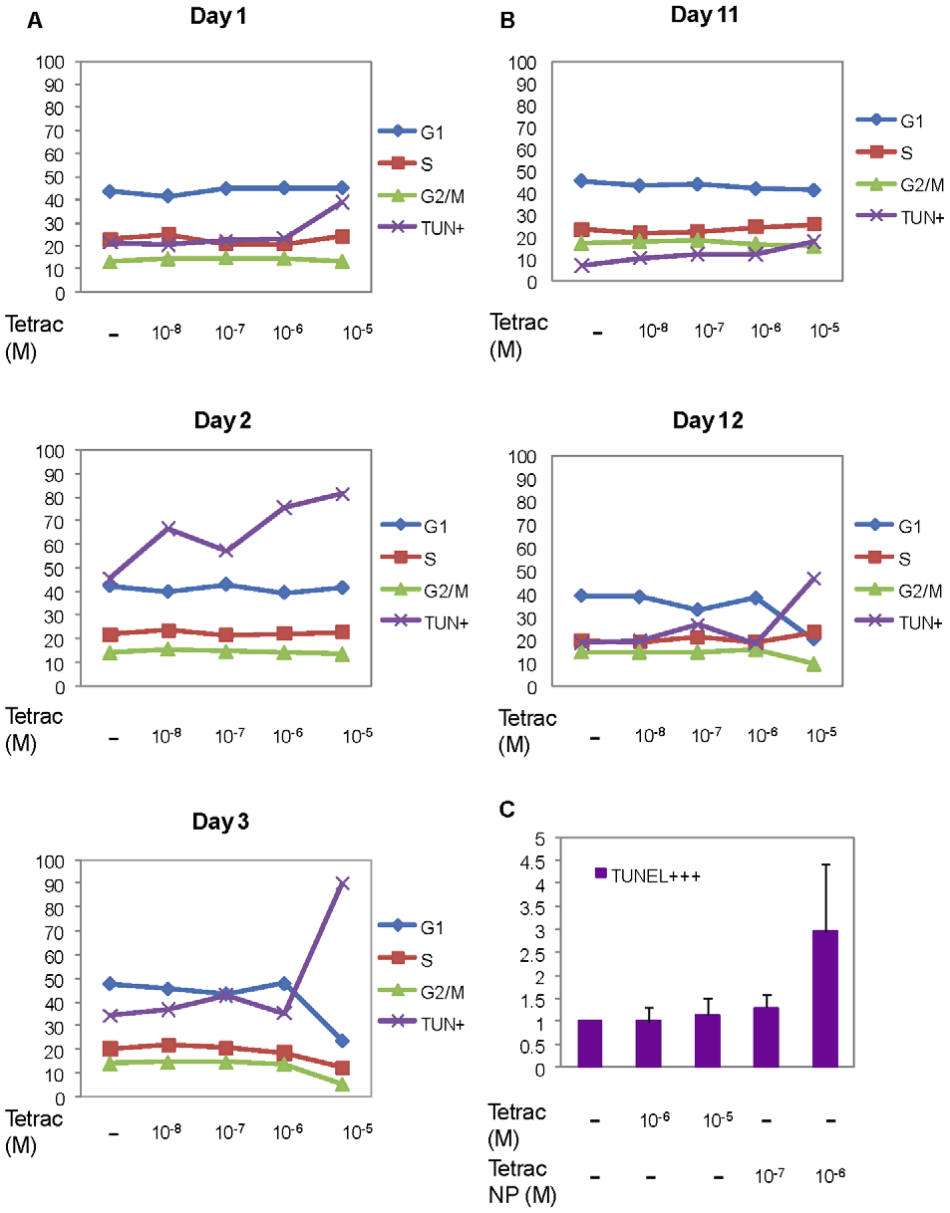
Tetrac induces apoptosis in MDA-MB-231 cells. (**A**) MDA-MB-231 cells grown in the perfusion bellows cell culture system were treated with different constant concentrations of tetrac (10^−7^ M to 10^−5^ M) for 12 d, and harvested on the days indicated. Two million cells from each sample were prepared for flow cytometry as described in the Materials and Methods section. Over 1–3 d treatment with these 4 concentrations of tetrac, the percentages of cells in G_1_, S or G_2_/M phases remained approximately the same, while TUNEL levels rose, particularly with the highest tetrac concentration, to 90% of the cells examined by day 3. (**B**) By days 11 and 12, the percentage of cells in phase G_1_ remained at approximately 40% except for cells exposed to the highest nano-tetrac concentration (20% in phase G_1_); TUNEL levels rose at the same concentration. Percentages of cells in G_2_/M and S phases were relatively constant. (**C**) Increases in TUNEL reactivity were not remarkable with either 10^−6^ or 10^−5^ M tetrac, whereas 10^−6^ M nano-tetrac caused a 3-fold increase in apoptosis. These results, obtained after exposure of cells to tetrac formulations for 3 d, confirm prior studies showing that nano-tetrac is more effective than tetrac at similar concentrations in causing changes consistent with a pro-apoptotic effect on cancer cells [8], [9].

Cells were harvested from the perfusion bellows cell culture system for flow cytometry analysis after 1–3 d of treatment with 10^−8^ to 10^−5^ M tetrac. There was a 1.8-fold increase of apoptotic cells with 10^−5^ M tetrac compared to untreated cells at 1 d (Fig. 6A). By days 2 and 3, all tetrac concentrations caused apoptosis, as determined by TUNEL assay. In cells continuously exposed to tetrac for more than 10 d, only 10^−5^ M tetrac produced apoptosis consistently (Fig. 6B), suggesting that tetrac may induce some cell proliferation, although the G_1_ phase was decreased by 50% after 12 d of tetrac treatment. The degree of apoptosis induced by 10^−6^ M nano-tetrac was 3-fold that of 10^−6^ M tetrac (Fig. 6C).

**Figure 7:**
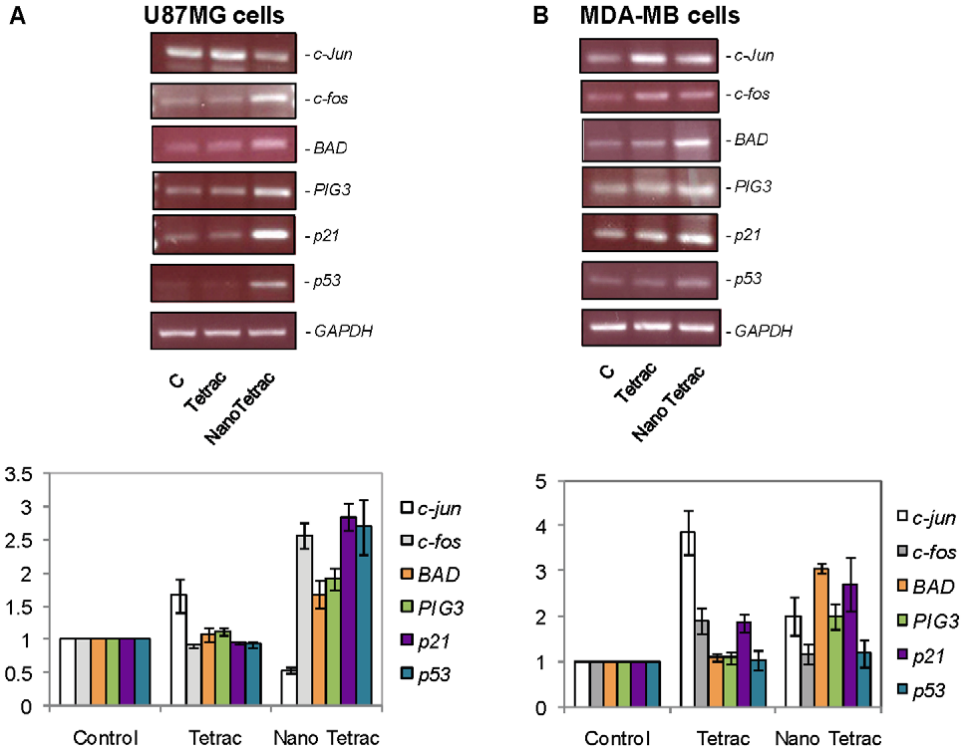
Expression of pro-apoptotic genes by tetrac and nano-tetrac. (**A**) U87MG human glioblastoma cells and (**B**) MDA-MB-231 breast cancer cells were treated with constant concentrations of 10^−6^ M tetrac or nano-tetrac in the perfusion bellows cell culture system. Cells were harvested after 2 d of treatment and total RNA was extracted. RT-PCR was carried out as described in the Materials and Methods section. Nano-tetrac significantly stimulated (P<0.02) the expression of pro-apoptotic genes (*p53*, *BAD*, *PIG3*, *p21*) [34], [35] in U87MG cells, while unmodified tetrac was effective only as an inducer of expression of *c-jun*. The results in MDA-MB-231 cells were different, in that tetrac enhanced the expression of *c-jun*, *c-fos* and *p21* (each, P<0.05 vs. control) to a moderate degree. Nano-tetrac induced expression of *BAD* (P = 0.001), *PIG3* (P = 0.037) and *p21* (P = 0.05). Together, results in the figure demonstrate the variable nature of responses to tetrac in the two cell lines and a more consistent response of each cell line to nano-tetrac.

We have recently reported that tetrac and nano-tetrac induce gene expression profile changes in MDA-MB cells [8] and medullary thyroid carcinoma cells [9]. Experiments presented here examined pro-apoptotic gene expression in tetrac- and nano-tetrac-treated glioblastoma U87MG cells and MDA-MB cells in the perfusion bellows cell culture system. RNA was extracted from the harvested cells at the end of treatment for RT-PCR studies. Treatment of cells for 2 d with nano-tetrac (10^−6^ M) increased expression of *PIG3*, *BAD*, *p21* and *p53* in both U87MG and MDA-MB cells (Fig. 7). In contrast, tetrac (10^−6^ M) did not significantly increase expression of this panel of genes in U87MG cells and, except for *c-jun*, gene expression in the MDA-MB cells was enhanced to a lesser extent by tetrac than by nano-tetrac. We have previously observed several differences between gene expression profiles that result from treatment with unmodified tetrac and nano-tetrac [9].

Experiments of flow cytometry and gene expression demonstrate the practicality of harvesting tumor cells from polymer flakes in the perfusion bellows cell culture system for studies of post-treatment states of the cells.

### Lack of effects of tetrac and nano-tetrac on non-malignant cells

We also determined whether tetrac and nano-tetrac had anti-proliferative actions on immortalized non-malignant cells. Monkey kidney epithelial CV-1 cells and human embryonic kidney 293T cells were treated daily with 10^−6^ M tetrac or 10^−6^ M nano-tetrac for 7 d. There was no significant change in cell numbers or morphology (results not shown here) when untreated cells were compared with those exposed to tetrac or nano-tetrac.

### Combined resveratrol and tetrac exposure and cancer cell proliferation

**Figure 8:**
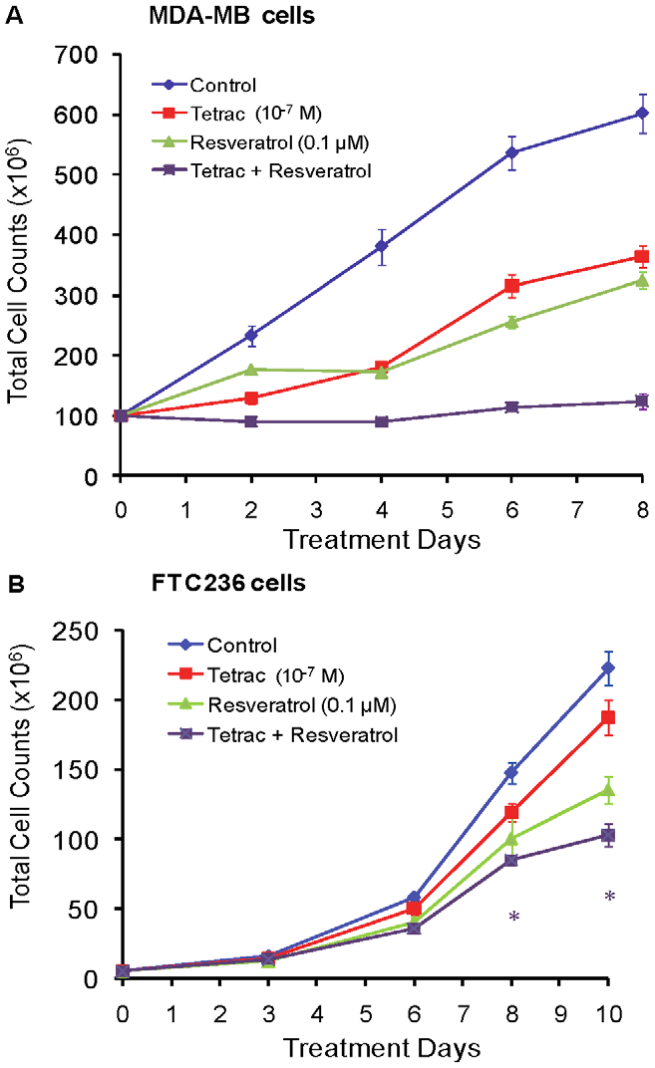
Effect of tetrac on resveratrol-induced anti-proliferation in human cancer cells. (**A**) Human breast cancer estrogen receptor-negative MDA-MB-231 cells were treated with constant concentrations of resveratrol (0.1 µM) and/or tetrac (10^−7^ M) in the perfusion bellows cell culture system. Cell aliquots were harvested daily for counting. Both agents individually caused suppression of cell proliferation and together they caused an additive effect. (**B**) Human follicular thyroid cancer FTC236 cells were treated daily with resveratrol (0.1 µM) and/or 10^−7^ M tetrac in the perfusion bellows cell culture system. Cell aliquots were again harvested daily for cell counting. At 8 and 10 d, based on both unadjusted and Holm t tests, the following results were obtained: tetrac+resveratrol, P<0.05 (unadjusted t test) and P = 0.066 (Holm t test). *, *P*<0.05 including α-adjustment for six comparisons (Holm t test). Multiple observations at each time point are multiple cell counts from one experiment.

A naturally-occurring stilbene, resveratrol [14], induces apoptosis in human follicular thyroid cancer cells [4], [17]. Thyroid hormone analogue T_4_ inhibits the apoptotic action of resveratrol [3], [4] and tetrac has been shown to restore the pro-apoptotic effect of the stilbene in presence of T_4_ [3]. This effect of tetrac reflects displacement by tetrac of T_4_ from the iodothyronine receptor site on integrin αvβ3. Resveratrol is capable of binding to the integrin αvβ3 [3], [18], at a site distinct from that for tetrac and other thyroid hormone analogues [3], [4]. In the present studies, the anti-proliferative effect of combined resveratrol and tetrac exposure was tested. Cancer cells were treated with resveratrol (0.1 µM) in presence or absence of 10^−7^ M tetrac. Both tetrac and resveratrol individually caused anti-proliferative effects in MDA-MB cells (Fig. 8A), while their combination was additive, based on comparison of cell counts on day 8 and Loewe additivity. Human follicular thyroid cancer (FTC) cells were treated daily with resveratrol (0.1 µM) in presence or absence of 10^−7^ M tetrac. Compared with breast cancer cells, FTC236 cells were less sensitive to tetrac (Fig. 8B). The inhibitory effects of resveratrol and tetrac in combination were additive also in FTC cells, based on cell counts on day 10.

### Tetrac increases the anti-proliferative action of cetuximab in human breast cancer cells

**Figure 9:**
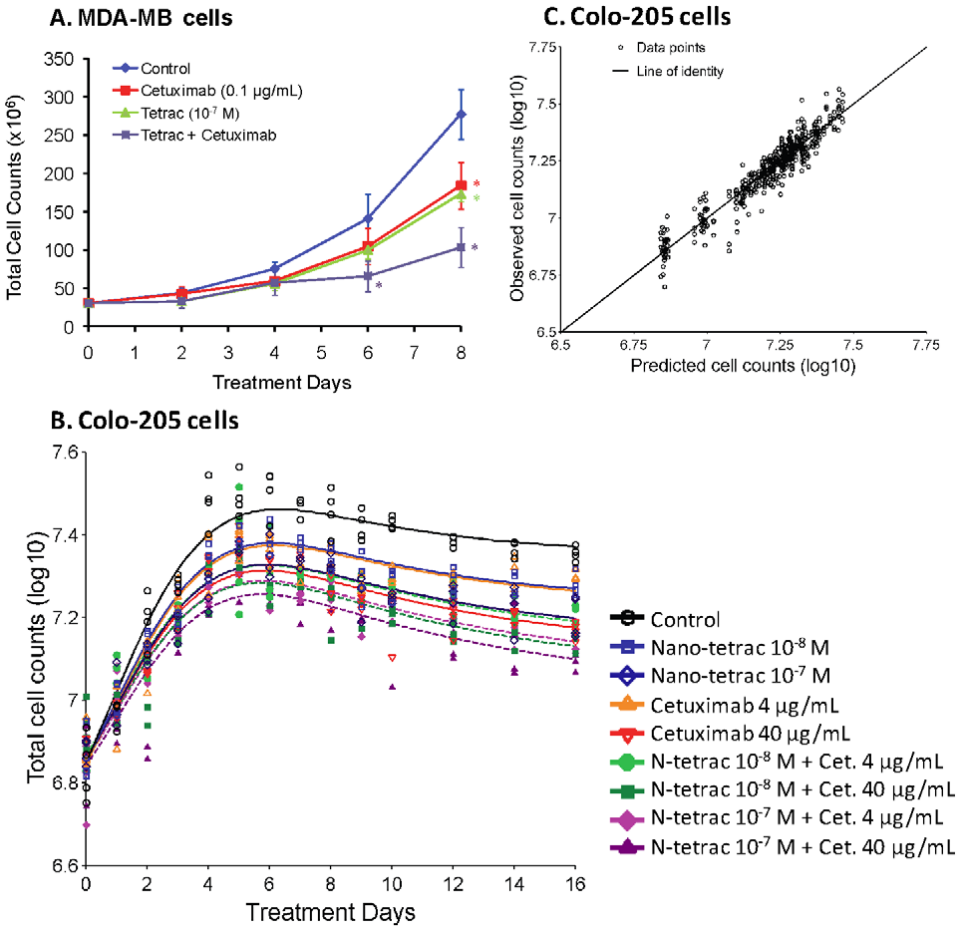
Effect of tetrac and nano-tetrac on cetuximab-induced anti-proliferation. (**A**) Human breast cancer MDA-MB-231 cells were treated with constant concentrations of 0.1 µg/mL of cetuximab in the presence or absence of 10^−7^ M tetrac in the perfusion bellows cell culture system for 8 d. Aliquots of cells were harvested and counted at the indicated time points. Levels of significance based on unadjusted t test (Holm t test) were the following: cetuximab, alone, vs. control at 6 d, P = 0.13 (0.13), and at 8 d, P = 0.006 (0.025); tetrac alone vs. control at 6 d, P = 0.052 (0.10), and at 8 d, P = 0.0004 (0.002); cetuximab + tetrac vs. control at 6 d, P = 0.008 (0.023), and at 8 d, P 0.0004 (0.002). *, *P*<0.05 including α-adjustment for six comparisons (Holm t test). Multiple observations at each time point are multiple cell counts from one experiment. (**B**) Colo 205 cells in cell culture flasks were treated with two different constant concentrations of nano-tetrac and cetuximab, alone or in combination. Multiple observations at each time point are multiple cell counts from one experiment. Lines are model fitted cell counts. (**C**) Observed versus predicted cell counts corresponding to the experiment and modeling shown in (B).

Cetuximab is a monoclonal antibody targeted to the extracellular domain of the EGFR intended for use in patients with metastatic colorectal carcinoma and certain other tumors [19], [20]. Effectiveness is variable [21], [22]. The drug has been combined clinically with various other chemotherapeutic agents in colorectal cancer patients [21], [22] and recently has been tested adjunctively *in vitro* against breast cancer cells [23]. Combining cetuximab with various chemotherapeutic agents has revealed additive or potentiated growth inhibition in various cancer cell lines [21], [22]. To determine whether tetrac potentiates cetuximab-induced anti-proliferation, human breast cancer MDA-MB cells were treated with cetuximab (0.1 µg/mL) in presence or absence of 10^−7^ M tetrac. Individually, both agents suppressed proliferation of MDA-MB cells (Fig. 9A). After 8 d treatment with cetuximab and tetrac the average total cell counts were decreased by 34% and 38%, compared to control. Combined tetrac and cetuximab was more effective, reducing total cell numbers on average by 63%. Application of an empirical mathematical model to all treatments and time points simultaneously also suggested an approximately additive effect of both compounds. The empirical model was a disease progression type model where the cell counts in the control treatment were described by a simple exponential function. The effect of tetrac was described as an offset, i.e. a change from baseline cell counts while tetrac is present. The effect of cetuximab was described in the same way, only including an additional lag-time of effect. When both drug effects were added the resulting profile adequately described the cell counts during combination treatment for the studied concentrations and observation period.

### Nano-tetrac increases the anti-proliferative action of cetuximab in colon cancer cells

An approximately additive effect was also found for the combination of nano-tetrac and cetuximab in human colon cancer Colo-205 cells (Fig 9 B). Colo-205 cells grown in T-75 flask were treated with either nano-tetrac (10^−8^ and 10^−7^ M), cetuximab (4 and 40 µg/ml), or combination. Medium was refreshed with agents daily. Cells were harvested and counted as indicated up to 16 days. A model including effects of both drugs on the probability of successful replication and a noncompetitive interaction adequately described the observed cell counts (Figs. 9B and 9C). The effect of the combination treatments was slightly larger than predicted by a competitive interaction model, where both drugs work on the same pathway, and slightly smaller than predicted by a purely noncompetitive interaction model, where the drug works on completely different pathways. Therefore a factor ψ was included (see equation in the Materials and Methods section) which was estimated at 5.6. The ImaxR and IC50R for inhibition of the probability of successful replication were 0.12 and 7.0 nM for nano-tetrac and 0.13 and 3.3 µg/mL for cetuximab.

## Discussion

Using a novel perfusion bellows cell culture system developed in our laboratory (Fig. 1), we have compared the pharmacodynamics *in vitro* of unmodified and nanoparticulate formulations of tetrac as anti-proliferative agents. The system revealed that nano-tetrac had a higher potency than tetrac as an anti-proliferative agent (Fig. 4). Neither nano-tetrac nor tetrac affected proliferation of two non-cancer cell lines even at high concentrations (10^−6^ M).

The anti-proliferative effect of tetrac and nano-tetrac on cancer cells in the perfusion bellows cell culture system was seen starting 3 d after start of treatment (Fig. 3, 4). The anti-cancer effects of tetrac and nano-tetrac in human tumor cell xenografts were well-established within 3 d after onset of drug administration [9]. These results in the perfusion system thus reproduce findings obtained earlier in cells grown in culture dishes and xenografts. While the tetrac effects in xenografts have been shown to involve both primary effects on tumor cell proliferation and an anti-angiogenesis effect [6], the effect of tetrac and nano-tetrac in the perfusion bellows cell culture system of course is limited to suppression of cell proliferation.

*In vitro* models such as described here can save animals by decreasing the number of animal studies which need to be conducted, by employing well-defined conditions which allow for investigation of individual factors impacting the PD and permitting the simulation of human pharmacokinetics (PK) based on data from clinical trials. Limitations of the method described here which need to be considered are that the impact of tissue penetration and the effect of the immune system are usually not directly taken into account; PK/PD models based on animal or clinical studies that include measurement of drug concentrations in tumor need to be developed.

In the perfusion system cells are exposed alternately to fresh medium and air. This paradigm optimizes growth conditions for cancer cells by maximizing nutrient uptake and oxygen transfer and supported experiments of up to 3 weeks' duration (Fig. 3B). Information obtained in longer studies about both the slope of the growth/proliferation phase and the plateau of the cell count with regard to time permitted mathematical modeling to identify two different effects of tetrac on cancer cells: inhibition of growth rate and inhibition of success of replication (Fig. 2).

In addition to treating the cells with constant drug concentrations, reflecting *in vivo* continuous infusion treatment, the *in vitro* system described here allows to study other dosing regimens. Multiple short-term or intermittent infusions or brief injections can be studied in the perfusion system by adjusting the flow rate of the medium and the dosing schedule. Drug concentration/time profiles that are expected or have been obtained in human or animal studies can be simulated and effects on cancer cells of changing drug concentrations as anticipated *in vivo* may be observed in the system. Together with mathematical modeling, these *in vitro* paradigms can support optimization of design of subsequent animal and human studies thereby saving time and expense. Because a wider range of drug concentrations can be studied *in vitro* than in animal models, dose selection for *in vivo* studies may become more efficient.

Mathematical modeling was utilized to increase the amount of information gained from the reported experiments. By considering the entire time course of cell counts in response to multiple concentrations of tetrac and control treatment simultaneously, more insight can be gained into the dose-response relationship and mechanism of action of a drug. Purely empirical growth models, e.g., the Weibull model, often do not include meaningful parameters, but offer arbitrary coefficients. For simulating other scenarios, e.g., cells with faster growth rates, mechanism-based models may be more adequate. While only total cell counts were available in the perfusion bellows cell culture system experiments reported here the applied model is based on mechanisms of action. Inclusion of flow cytometry results in the model will be performed for future experiments in order to enhance the mechanism-based modeling approach.

For U87MG cells studied here, mathematical modeling suggested a higher maximum effect but lower sensitivity of the effect on probability of successful replication, compared to the effect on growth rate for both unmodified and nano-tetrac. For both effects the sensitivity favored nano-tetrac over unmodified tetrac. This may be explained by the ability of unmodified tetrac to penetrate into cells and thereby exert low-grade thyromimetic (proliferative) effects in addition to the anti-proliferative effects initiated at the cell surface integrin receptor. Therefore the net anti-proliferative effect of unmodified tetrac is decreased. The model describes the net effects of unmodified tetrac. Nano-tetrac does not gain access to the cell interior and shows a more robust anti-proliferative effect.

MDA-MB cells had growth rate sensitivity to nano-tetrac that was similar to unmodified tetrac, but a higher sensitivity to nano-tetrac for the effect on success of replication. For both unmodified tetrac and nano-tetrac MDA-MB cells were more sensitive to the effect on success of replication than the effect on growth rate. The uncertainty of the parameter estimates was explored by bootstrap runs. A very rich sampling design was used to ensure the general estimability of the model by two different algorithms. In addition, the estimability was tested under the sampling designs of the actual experiments. For the models of unmodified tetrac, the 10% percentile to 90% percentile intervals (P10–P90) were relatively narrow. A larger uncertainty was seen for the IC50 parameters in the nano-tetrac models, especially for the IC50k in MDA-MB cells. The latter suggests that the effect on rate of growth was not apparent in all of the randomly created bootstrap datasets. Optimal design was not applied to structuring those experiments but will be utilized in future studies. It is important to note that the studied concentrations were 10-fold different between the treatment arms and, based on that factor, the uncertainties in IC50 and the differences in the estimates between NONMEM and S-ADAPT are acceptable. Overall our mechanism-based models have adequately described the cell counts over time in our studies and the effects of a wide range of tetrac concentrations and will support the design of future experiments. In addition to the pharmacodynamic studies *in vitro* and in animals, also the pharmacokinetics of tetrac will be studied *in vivo* to more fully characterize the pharmacokinetic / pharmacodynamic relationship for tetrac *in vivo*.

We have previously shown that resveratrol induces apoptosis in human cancer cells, an effect which requires the nuclear translocation of COX-2 and activated ERK1/2 for support of p53-dependent apoptosis [3], [17]. Resveratrol and tetrac both bind to plasma membrane integrin αvβ3 [1], [18], but at discrete sites that apparently do not interfere with one another [3], [7]. In the present studies, the combination of resveratrol and tetrac was additive in the *in vitro* perfusion bellows cell culture system in terms of suppression of cell proliferation in two human cancer cell lines. The ability to detect such additivity—or potentiation, if present—is obviously a requirement of the perfusion system.

Therapeutic epidermal growth factor receptor (EGFR) targeting with cetuximab, either as single agent or in combination with chemotherapy, has demonstrated variable clinical activity [19] and may benefit only select patients [20]. In the perfusion bellows cell culture system, concurrent treatment with tetrac and cetuximab resulted in highly effective inhibition of proliferation of MDA-MB cells by day 8 (Fig. 9A). The model system thus offers the prospect of efficiently exploring a variety of drug combinations. An empirical disease progression model was employed for the combination treatment of MDA-MB cells with tetrac and cetuximab, and revealed an approximately additive effect for the combination. While such an empirical model has limitations it is not feasible to develop a receptor occupancy model for a drug combination without data at multiple drug concentrations. Two concentrations each of nano-tetrac and cetuximab and all four combinations were studied in Colo-205 cells in cell culture flasks. The effects of nano-tetrac and cetuximab were adequately described as inhibition of the probability of successful replication. Modeling of all treatment arms simultaneously revealed an approximately additive effect of the combination. The effect of the combination treatment was slightly smaller than predicted by a purely noncompetitive interaction and slightly larger than predicted by a purely competitive interaction model. This suggests that there is a partial overlap between the mechanisms and pathways of action of nano-tetrac and cetuximab. That interpretation of the modeling results is supported by previous studies in our laboratory where we showed that tetrac interferes with crosstalk between the cell surface receptor for thyroid hormone and EGFR [24] and it can be assumed with confidence that nano-tetrac also interferes with this crosstalk. In addition, nano-tetrac, but not unmodified tetrac, decreases the expression of the EGFR gene [8]. For this study in cell culture flasks it was observed that cell counts in all treatment arms decreased noticeably and approximately in parallel after Day 6 (Fig 9B) which cannot be attributed to drug effect. Such observations further support the use of the perfusion bellows cell culture system which provides optimal nutrient uptake and oxygen transfer for the cells and will be utilized for future combination studies in colon cancer cells.

The perfusion bellows cell culture studies we described provide useful pharmacodynamic information on the application of new drugs or combinations of various agents *in vitro* to human cancer cell lines. In combination with pharmacodynamic modeling and by including information about the expected pharmacokinetics of a drug, the perfusion bellows cell culture system permits study of the dose-response relationships of anti-neoplastic agents over a very wide concentration range *in vitro*, and can support translation from *in vitro* models to animal models and human clinical trials.

## Materials and Methods

### Cell lines

Human glioblastoma cells (U87MG), human breast cancer MDA-MB-231 cells (MDAMB), human colon cancer Colo-205 cells, African green monkey kidney epithelial CV-1 cells and human embryonic kidney 293T cells were purchased from ATCC. Human follicular thyroid cancer FTC236 cells were generously provided by Dr. Orlo Clark (University of California at San Francisco-Mt. Zion Medical Center, San Francisco, CA). U87MG cells were maintained for study in MEM (Gibco, Carlsbad, CA) supplemented with 10% fetal bovine serum (FBS, Sigma Aldrich, St. Louis, MO). Colo-205 cells were maintained in RPMI (Gibco) supplemented with 10% FBS. MDA-MB, CV-1 and 293T cells were maintained in DMEM (Gibco) supplemented with 10% FBS. Follicular thyroid cancer cells were supported in 50% DMEM/50% Ham's F-12 (Gibco) plus 10 mU/ml of TSH (Sigma Aldrich). Cells were cultured in a 5% CO_2_/95% air incubator at 37°C.

### Pharmacodynamics (PD) of tetrac

Shown in Fig. 1 is a newly developed perfusion bellows cell culture system that is a disposable bioreactor capable of high density cell culture for studies of anti-cancer drugs. Each cell culture system is a compressible (bellows) 500 mL bottle that contains cell culture medium and specially-treated polymer flakes to which cells spontaneously attach and then proliferate. Through moving bellows and porous membranes the level of the medium in the bottle changes periodically. Consequently, the cells are alternately submerged in the culture medium and exposed to 5% CO_2_/95% air which creates a dynamic interface between air and medium on the plated cell surface that maximizes nutrient uptake and oxygen transfer. The system provides a low shear, high aeration and foam-free culture environment. Proprietary treatment of the surfaces of the flakes enables seating and the harvesting of cells and secreted proteins are readily isolated from the perfusate.

In a non-perfusion bellows cell culture system that was also used, the medium in each bottle was replaced by fresh medium every 24 h. In the perfusion bellows cell culture system, medium was progressively refreshed over 24 h, so that one complete change of medium occurred over 24 h.

To establish the cultures, 5×10^7^ cells were seeded in perfusion and non-perfusion bellows bottles and incubated overnight at 37°C. Flakes were then harvested, trypsinized, and the cells were collected and counted. The number of cells that attached to flakes was 10–15×10^6^ per bottle. For experiments, the perfusion bellows cell culture system was run for 2 d prior to starting the experiments. The cell numbers at this point were about 30–50×10^6^ cells per bottle. Cultured cells were then exposed to 1% FBS-containing medium. Tetrac or nano-tetrac was added to the medium in the reservoir bottle to achieve the final concentrations reported for each experiment.

Nano-tetrac utilized in the studies of proliferation of MDA-MB, U87MG, and Colo-205 cells was manufactured on contract by Azopharma (Miramar, FL). Nano-tetrac for all other experiments was prepared at the Pharmaceutical Research Institute, Rensselaer, NY [9]. Unmodified tetrac was synthesized on contract by Peptido GmbH (Bexbach, Germany).

### Liquid chromatography–tandem mass spectrometry (LC/MS/MS)

In LC/MS/MS experiments, medium samples (20 µL) were injected onto an HP 1100 series HPLC system (Agilent Technologies, Palo Alto, CA, USA), equipped with a narrow-bore Zorbax Eclipse XDB-C18 column (5 µm, 150×2.1 mm; Agilent). Separation was performed using a mobile phase of 0.1% (v/v) acetic acid (A) and 100% acetonitrile (B), with a linear gradient of 20–60% B over 25 min. Flow rate was maintained at 0.2 mL min^−1^ and elution was monitored by a diode array detector (200–600 nm). The LC effluent was then introduced into a turbo ion-spray source on a Q/STAR-XL quadruple/time-of-flight (TOF) hybrid mass spectrometer (Applied Biosystems, Foster City, CA, USA). Negative ESI mass spectra were acquired over the range m/z 100 to 400. The electrospray voltage was set at −4.5 kV and the source temperature was maintained at 475°C. CID spectra were acquired using nitrogen as the collision gas under collision energies of 25–55 V. High purity nitrogen gas (99.995%) was used as the nebulizer, curtain, heater and collision gas source.

### RT-PCR

Total RNA was isolated as described previously [25]–[27]. First strand complementary DNAs were synthesized from 1 µg of total RNA, using oligo dT and AMV Reverse Transcriptase (Promega, Madison, WI). First-strand cDNA templates were amplified for *GAPDH*, *c-fos*, *PIG3*, *c-Jun*, and *BAD* mRNAs by polymerase chain reaction (PCR), using a hot start (Ampliwax, Perkin Elmer, Foster City, CA). Primer sequences were *GAPDH* (5′-AAGAAGATGCGGCTGACTGTCGAGCCACA-3′ [forward] and 5′- TCTCATGGTTCACACCCATGACGAACATG-3′ [reverse), *c-fos* (5′-GAATAAGATGGCTGCAGCCAAATGCCGCAA-3′[forward] and 5′-CAGTCA-GATCAAGGGAAGCACAGACATCT-3′ [reverse]), *PIG3* (5′-TGGTCACAG-CTGGCTCCCAGAA-3′ [forward] and 5′-CCGTGGAGAAGTGAGGCAGAATTT-3′ [reverse]), *c-jun* (5′-GGAAACGACCTTCTATGACGATGCCCTCAA-3′ [forward] and 5′-GAACCCCTCCTGCTCATCTGTCACGTTCTT-3′ [reverse) and *BAD* (5′-GTT-TGAGCCGAGTGAGCAGG-3′ [forward] and 5′-ATAGCGCTGTGCTGCCCAGA-3′ [reverse]). The PCR cycle was an initial step of 95°C for 3 min, followed by 94°C for 1 min, 55°C for 1 min, 72°C for 1 min, then 25 cycles and a final cycle of 72°C for 8 min. PCR products were separated by electrophoresis through 2% agarose gels containing 0.2 µg of ethidium bromide/mL. Gels were visualized under UV light and photographed with Polaroid film (Polaroid Co., Cambridge, MA). Photographs were scanned under direct light for quantitation and illustration. Results from PCR products were normalized to the GAPDH signal.

### Flow cytometry analysis

Cells were harvested from flakes by trypsinization, washed with PBS, fixed in ice-cold 70% ethanol and stored in a freezer overnight. Cells were labeled to detect apoptosis with the *In situ* Cell Death Detection Kit, Fluorescein (Roche Diagnostics Corporation, Roche Applied Science, Indianapolis, IN). The recommended procedures were used with modifications in permeabilization time and temperature to improve results. Fixed cells were centrifuged and washed once in PBS containing 1% bovine serum albumin (BSA), then resuspended in 2 mL permeabilization buffer (0.1% Triton X-100 and 0.1% sodium citrate in PBS) for 25 min at room temperature, followed by a wash in 0.5 mL PBS/1% BSA. Cells were resuspended in 50 µL TUNEL reaction mixture (TdT enzyme and labeling solution) and placed in an incubator for 60 min at 37°C in a humidified dark atmosphere. Labeled cells were washed in PBS/1% BSA, then resuspended in 0.5 mL ice-cold PBS/0/1% BSA Triton X-100 that contained 1 µg/mL 4′, 6-diamidino-2-phenylindole (DAPI) for at 20 min. Cell samples were analyzed with a BD™ LSR II (BD Biosciences, San Jose, CA), using BD FACSDiva™ software. Fluorescence histograms were gated on forward scatter (FSC) and side scatter (SSC) to exclude debris and clumped cells. Gating on height vs. area fluorescence of DAPI signal was set to eliminate clumped cells and to obtain the singlet population for analyzing the cell cycle phase ratios in G_1_, S or G_2_/M.

### Statistical analysis

Immunoblot and nucleotide densities were measured with a Storm 860 phosphorimager, followed by analysis with ImageQuant software (Molecular Dynamics, Sunnyvale, CA). Student's *t* test, with P<0.05 as the threshold for significance, was used to evaluate the significance of the hormone and inhibitor effects. Where cell counts were tested for statistical significance, the data were log-transformed prior to testing. For the cell count data, an α-adjustment to account for multiple comparisons was utilized according to the Holm *t* test. The concept of Loewe additivity [28] was applied to cell count data from combination treatments. For experiments involving cells counts at many time points, for multiple treatments, or both, multiple *t* tests were not an adequate method of analysis due to the large number of comparisons. In addition, multiple comparison tests treat the observations at each time point independently, whereas mathematical modeling, as described below, takes into account the full time course. Observed data are presented in the figures as individual data points or average ± standard deviation (SD).

### Mathematical modeling

The time course of cell counts of the several cancer cell lines treated with different concentrations of tetrac or nano-tetrac (or a combination of tetrac with cetuximab or resveratrol, or nano-tetrac with cetuximab) was modeled utilizing a naïve pooled approach in NONMEM VI (version 6.2). The pooled approach does not distinguish any potential unexplained variability between the bottles (treatment arms) from general assay error, e.g., uncertainty in cell counts, but expresses both in the residual error. The perfusion bellows cell culture system experiments included one bottle per treatment arm with the multiple observations per time point being different cell counts of one sample for the tetrac experiments and the nano-tetrac with cetuximab combination study, and average cell counts from three studies for the nano-tetrac experiments. The population approach in NONMEM (FOCE) did not succeed in distinguishing inter-subject variability (variability between bottles) and unexplained random variability (e.g. general assay error). The naïve pooled analysis in NONMEM was equivalent to a pooled analysis using the Maximum Likelihood approach in ADAPT, for example. S-ADAPT was also utilized as described below in order to make use of the MC-PEM algorithm and for additional model evaluation. All time points and treatment arms within each experiment were modeled simultaneously. A mechanism-based model [29] was adapted to describe the proliferation of cancer cells and the inhibition of proliferation by tetrac. This model assumes two populations of cells in different phases of the cell cycle: cells that are preparing for replication (phase 1) and cells that are immediately ‘pre-replication’ (phase 2). Cells transition from phase 1 to phase 2 by a first-order growth rate constant, while replication from phase 2 to phase 1 is assumed to be fast (Fig. 2).

The number of cells in phase 1 and 2 are described by:
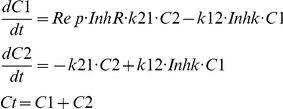
where C1 is the number of cells in phase 1, C2 the number of cells in phase 2, k21 the first order rate constant for replication (transition from phase 2 to phase 1), and k12 the first-order growth rate constant for transition from phase 1 to phase 2. The k21 was assumed to be fast and therefore was fixed to 100 day^−1^, which resulted in a ratio of k21/k12 of approximately 50 to 100, depending upon the cell line. The total number of cells Ct is the sum of C1 and C2. Rep is the replication efficiency factor which is described by:
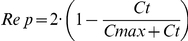
where Cmax is the maximum number of cells. Without tetrac (or nano-tetrac), the replication efficiency factor approaches 2, which reflects a 100% probability of successful replication. When Ct approaches Cmax, Rep approaches 1, representing a 0% probability of net replication, that is, cells in reality still transition between the phases, but the number of cells does not increase further. The InhR describes the inhibitory effect of tetrac on the probability of successful replication:
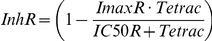
where ImaxR is the maximum effect of tetrac (or nano-tetrac) on the probability of successful replication and IC50R is the tetrac concentration needed to achieve a half-maximal effect. In the case of InhR< 0.50, this effect results in cell killing, as it then follows that Rep • InhR< 1.0. The latter case also illustrates that cells which do not replicate successfully are eliminated in this process. For some studies, inclusion of a decrease in ImaxR over time was necessary in order to adequately describe the data:
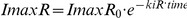
where ImaxR_0_ is the ImaxR at time = 0 and kiR is a constant describing the decrease of ImaxR over time.

Inhk describes the inhibitory effect of tetrac on the rate of growth:
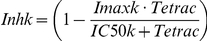
where Imaxk is the maximum effect of tetrac on rate of growth and IC50k is the tetrac concentration needed to achieve a half-maximal effect. Both IC50R and IC50k are measures for the sensitivity of the cancer cells to the effects of tetrac. A low IC50 corresponds to a high sensitivity of the cells to a particular drug effect, and vice versa. While the InhR describes an irreversible removal of cells from the cell cycle, Inhk only slows down the transitioning of cells through the cell cycle. The cells remain in state 1 for a longer period of time which represents growth and preparation for replication. This is reflected in a decreased slope of the growth curve.

Although cells in state 1 and state 2 were not measured separately in the perfusion bellows cell culture system experiments reported here, the two effects were distinguishable and the parameters estimable. The effect on rate of growth decreases the slope of the growth curves whereas the effect on successful replication results in lower plateaus at the end of the growth curves for the treatment arms compared to control. As described below simulation estimation runs were performed to confirm the estimability of the parameters.

The effects of nano-tetrac were modeled by the same equations as described above for unmodified tetrac. However the IC50 estimates for nano-tetrac are hypothetical concentrations that assume all of the tetrac bound to the nanoparticle is available for binding to the integrin receptor.

A lag time for growth was included in order to describe the data successfully. The parameter k12 was low at the start of the experiment and increased over time:
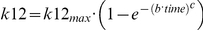
Here, k12_max_ is the maximum growth rate constant and b and c are empirical constants. The residual variability was described by an additive error on log-scale.

A model for non-competitive interaction was applied to the experiment on the effects of nano-tetrac, cetuximab, and their combination on Colo-205 cells. The effects of nano-tetrac (InhR_NPT_) and cetuximab (InhR_CET_) were described as:
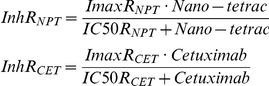
The effect of the combination was:
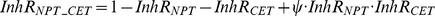
which describes a non-competitive interaction [30], [31] when ψ = 1, that is both drugs act by completely separate pathways [32], [33]. When ψ>1, then the effect of the combination is less than would be expected from two drugs acting completely independent of each other. The decrease of cell counts in all treatment arms towards the end of the observation period in this study in cell culture flasks was modeled by a series of transit compartments.

Model discrimination was based on comparison of the objective function in NONMEM, visual comparisons of observed and fitted cell counts over time, and observed vs. fitted plots. Simulation estimation experiments (bootstraps) were performed for the models of tetrac and nano-tetrac effects on U87MG and MDA-MB cells in order to explore the estimability of the model and the bias and uncertainty in the parameter estimates. The simulations were done in Berkeley Madonna (v.8.3.14). The estimations were performed in both NONMEM (pooled approach) and the MC-PEM (Monte Carlo parametric expectation maximization) algorithm in parallelized S-ADAPT (v.1.56). One hundred bootstrap datasets in NONMEM and fifty bootstrap datasets in S-ADAPT, each with 10 profiles per treatment arm, were run for each of the four experiments (two cell lines and two formulations), assuming a very rich sampling schedule and an additive error on log-scale of 0.1 (Bootstraps based on additive errors on log-scale of 0.02, 0.05, and 0.1 had been previously conducted for the model of tetrac effects in U87MG cells). As the bootstraps were performed in order to obtain a point estimate for the parameters and not to characterize their distribution, and also due to long run times, 50 to 100 bootstrap runs each were adequate. Those bootstraps based on the rich sampling schedule were conducted to evaluate the mathematical estimability of the model parameters under ideal experimental conditions, i.e. many sampling time points. One hundred bootstrap datasets each with 10 profiles per treatment arm were run in NONMEM for each of the four models with the sampling schedules that were actually used in the experiments and assuming an additive error on log-scale of 0.1. The bootstraps based on the actual sampling schedules were performed to test whether the model parameters were well-estimable based on both the model and the experimental conditions. The median and 10% and 90% percentiles were calculated from each of those simulation estimation experiments.
